# A phase I study to evaluate the safety, tolerability, and pharmacokinetics of a novel, potent GABA analog HSK16149 in healthy Chinese subjects

**DOI:** 10.3389/fphar.2023.1296672

**Published:** 2023-12-11

**Authors:** Qian Chen, Qingqing Wu, Rong Song, Yating Wang, Mengqi Zhang, Fangqiong Li, Weifang Zeng, Wei Wang, Jingying Jia, Chen Yu, Yanmei Liu

**Affiliations:** ^1^ Drug Clinical Trial Center, Shanghai Xuhui Central Hospital, Shanghai, China; ^2^ Shanghai Engineering Research Center of Phase I Clinical Research and Quality Consistency Evaluation for Drugs, Shanghai, China; ^3^ Shanghai Institute of Clinical Mass Spectrometry, Shanghai, China; ^4^ Haisco Pharmaceutical Group Co., Ltd., Chengdu, China

**Keywords:** HSK16149, GABA analog, safety, pharmacokinetics, healthy subjects

## Abstract

**Purpose:** HSK16149 is a novel, potent gamma-aminobutyric acid (GABA) analog for the treatment of neuropathic pain. This study aimed to evaluate the safety, tolerability, and pharmacokinetics of HSK16149 after single and multiple doses in healthy Chinese subjects.

**Methods:** The randomized, double-blind, placebo-controlled study comprised two parts: SAD (single ascending-dose study) and MAD (multiple ascending-dose study). A total of 122 healthy subjects were enrolled in this study. HSK16149 capsule or placebo was administered as the protocol required. The safety of the drug was evaluated through clinical examinations and adverse events. Blood and urine samples were collected at the designated time intervals for pharmacokinetic analysis.

**Results:** Subjects were generally well tolerated after HSK16149 administration and the most common treatment-emergent adverse event (TEAEs) was dizziness, which was expected based on the mechanism of action of HSK16149. In SAD, AUC and C_max_ were shown to have a dose-proportional relationship in the dose range of 5-120 mg. The t_1/2_ of HSK16149 is 3.7-6.4 h. In MAD, after a single and multiple administration of 15-80 mg, AUC and C_max_ are proportional to the increased dose of HSK16149, and the accumulative ratios of AUC and C_max_ at steady-state were 1.05–1.44 and 1.07–1.36, respectively, indicating that HSK16149 only accumulated slightly after repeated administration.

**Conclusion:** HSK16149 was well tolerated in healthy Chinese subjects. Based on the safety and pharmacokinetic data, 80 mg twice daily (BID) was suggested as the highest target dose for further clinical development.

**Clinical Trial Registration:** http://www.chinadrugtrials.org.cn, identifier CTR20182535 and CTR20191317

## 1 Introduction

Diabetic Peripheral Neuropathy Pain (DPNP) exists in nearly 25% of diabetes patients, which will lead to impairment of daily functions, sleep disorders, depression, economic instability, and decline in quality of life (Stewart et al., 2007; Alleman et al., 2015). Due to the diversity of causes, its pathogenesis has not yet been fully elucidated (Jiang et al., 2013). There are several guidelines for the drug treatment of DPNP, all of which recommend gabapentin drugs (gabapentin, pregabalin) as appropriate first-line treatment methods (Pop-Busui et al., 2017; Bril et al., 2018; Ziegler et al., 2021; Blonde et al., 2022; Price et al., 2022; Ziegler et al., 2022). Gabapentin and pregabalin belong to the category of α2δ ligands that exhibit high-affinity binding to the extracellular α2δ subunit of voltage-gated calcium channels (Taylor, 2009; Sloan et al., 2022). The α2δ proteins are primarily expressed in the central nervous system (brain and spinal cord), and the regulation of these channels leads to a decrease in the release of excitatory neurotransmitters (Taylor, 2009). HSK16149 is an oral GABA analog with independent intellectual property rights developed by Haisco Pharmaceutical Co., Ltd. Its mechanism of action is similar to that of pregabalin. It can bind to calcium ion channel α2δ sub-receptors and reduce the calcium ion influx in voltage-dependent calcium channels in the central nervous system, thereby reducing the release of excitatory neurotransmitters such as glutamate, norepinephrine and substance P. It is expected to have antiepileptic, analgesic, and anti-anxiety activities (Bhusal et al., 2016; Shin et al., 2018). In preclinical studies, HSK16149 demonstrated greater potency and had a lower minimum effective dose compared to pregabalin (Gou et al., 2021). The duration effect of HSK16149 was also longer than that of pregabalin when both were tested at the same dose level, and its safety in the central system is better than that of pregabalin (Gou et al., 2021). In the phase II/III study conducted in China on DPNP patients aged ≥18 years, HSK16149 rapidly improved the analgesic effect without titration, and was effective and well tolerated for alleviating pain in Chinese DPNP patients (GUO et al., 2023).

In China, the current treatment of DPNP is still unsatisfactory. Although gabapentin drugs have been proven effective and recommended as first-line treatment methods, the indications for DPNP of pregabalin and mirogabalin have not yet been approved for marketing in China, possibly due to their side effects. There is an urgent need for some novel α2δ ligand drugs to treat DPNP patients. Due to its superior therapeutic index compared to pregabalin in preclinical studies, HSK16149 will undoubtedly has great potential to bring significant clinical benefits to domestic DPNP patients. Therefore, we conducted this fist-in-human phase I study to evaluate the safety, tolerability, and pharmacokinetics of HSK16149 after single and multiple administrations in healthy subjects.

## 2 Materials and methods

The study consisted of two parts: SAD (single ascending-dose study) and MAD (multiple ascending-dose study), both of which were randomized, double-blind, placebo-controlled studies.

The study was conducted at the Phase I Clinical Research Center of Shanghai Xuhui Central Hospital (Shanghai, China) from December 2018 to January 2020 and registered on http://www.chinadrugtrials.org.cn (Registration No. CTR20182535 and CTR20191317). Ethical approval for the study protocol was obtained from the Ethics Committee of Shanghai Xuhui Central Hospital [Ethical approval number: (2018) Ethics Review (051) and (2019) Ethics Review (016)]. The study was conducted in accordance with the Declaration of Helsinki and Good Clinical Practice. All subjects were required to provide written informed consent before any study-related procedures were performed.

### 2.1 Study design

**FIGURE 1:**
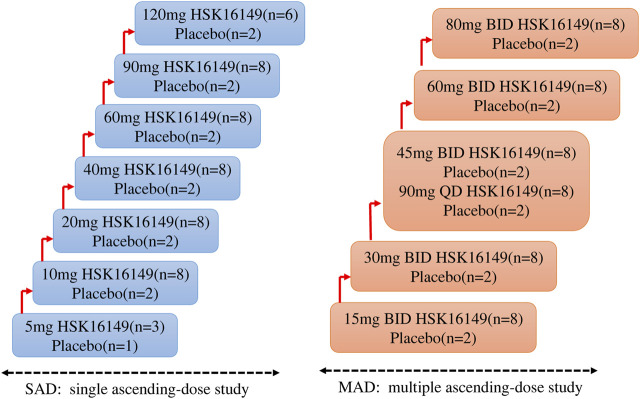
The scheme of study design.

SAD: A total of 62 subjects were involved in this part. Subjects were randomly assigned to one of the seven groups receiving placebo or HSK16149 at doses of 5, 10, 20, 40, 60, 90, and 120 mg. 4 (3 for HSK16149 and 1 for placebo) subjects were assigned to the starting dose of 5 mg group, 8 (6 for HSK16149 and 2 for placebo) for the highest dose group of 120 mg, and 10 (8 for HSK16149 and 2 for placebo) for the remaining dose groups (Figure 1).

Eligible subjects were admitted to the Phase I unit 1 day prior to drug administration. On the morning of Day 1, after a period of fasting for ≥10 h, subjects were administered HSK16149 or placebo at the respective dose level with 240 mL of water. All subjects stayed at the Phase I unit until 48 h after dosing with close medical monitoring. A follow-up visit by telephone or WeChat was performed by investigators on Day 7.

MAD: A total of 60 subjects were involved in this part. Subjects were randomly assigned to one of the six groups receiving HSK16149 (*n* = 8 per dose group) or placebo (*n* = 2 per dose group)at doses of 15, 30, 45, 60, and 80 mg, twice a day (BID), and of 90 mg once a day (QD) (Figure 1). The dose group 45 mg BID and 90 mg QD are parallel dose groups, that means when the tolerance assessment of the previous dose group is completed and the results show good tolerance, they can be carried out at the same time. Eligible subjects were admitted to the Phase I unit one day prior to the first administration. On the morning and evening (excluding the 90 mg QD group) of Day 1–8, HSK16149 capsule or placebo at the respective dose level was administered with 240 mL of water under fasting conditions. All subjects stayed at the Phase I unit until 48 h after the last dose. A follow-up visit by telephone or WeChat was performed by investigators on Day 15. This study initiated from the lowest dose group, and the decision to proceed to the next higher dose group was made by both investigator and sponsor according to the safety and tolerability of the preceding dose group within 7 days after the last administration or terminate according to the dose-escalation termination criteria specified in the protocol.

Randomization in this study was carried out using a stratified block randomization method. The double-blind study uses identical placebos corresponding to different specifications of HSK16149, with identical labels and outer packaging, ensuring that both subjects and researchers are completely unaware of the distribution of the experimental drug.

### 2.2 Participants

Healthy Chinese males or females aged between 18 and 45 years were erolled if they met the following inclusion criteria, but did not meet the exclusion criteria.

Inclusion criteria: bodyweight ≥50 kg for males, or ≥45 kg for females, and body mass index (BMI) between 19 and 26 kg/m^2^ (included); vital signs, physical examination, 12-lead ECG, chest X-ray and results of laboratory tests was normal or abnormality with no clinical significance; willing to use effective contraceptive measures during the study and within 3 months after the administration of investigational products.

Exclusion criteria: history of any systemic disorders or diseases, orthostatic hypotension, allergy, angioedema or any disease history or current disease that may affect the safety evaluation of the subject or the process of the study drug *in vivo*; clinically significant dizziness or vertigo; people with insomnia, anxiety disorder, depression disorder, or other mental disorders; any drug that inhibits or induces liver drug-metabolizing enzymes used within 30 days before the first administration of the study drug; blood donation or blood loss ≥400 mL within 3 months before dosing; any concomitant medication within 2 weeks before screening; participation in any clinical trials within 3 months before dosing; drug or alcohol addicts, or heavy smokers; positive test for hepatitis B, hepatitis C, HIV, or syphilis; positive pregnancy test for females.

### 2.3 Safety assessment

The safety of the study was evaluated through vital signs (blood pressure, pulse rate, respiratory rate, and body temperature), physical examinations, clinical laboratory tests, 12-lead ECGs, and adverse events (AEs) monitoring throughout the study. AEs were evaluated according to the National Cancer Institute Common Terminology Criteria for Adverse Events (CTCAE, version 5.0), and were processed and recorded promptly by qualified investigators according to relevant regulations. All AEs were coded using the Medical Dictionary for Regulatory Activities (MedDRA, version 22.0). The incidence of AEs was calculated by dividing the number of subjects who have experienced at least one AE by the total number of subjects in the corresponding part.

The dose-escalation termination criteria in the whole study were determined by AEs. Dose escalation should be terminated when 1) AEs related to the study drug of CTCAE Grade 2 and above occur in ≥50% of the subjects in a certain dose group or AEs related to the study drug of CTCAE Grade 3 and above occur in ≥33.3% of the subjects in a certain dose group; 2) Serious adverse events (SAEs) related to the study drug occur in at least one subject; 3) The drug exposure level shows a saturation trend and is approaching a plateau.

### 2.4 Biological sample collection

Blood samples (approximately 3 mL each) were collected from a suitable forearm vein by an indwelling catheter or by immediate venipuncture. In the 5 mg group of SAD, blood samples were collected at the following 16-time points: 0 (pre-dose), 0.25, 0.5, 0.75, 1, 1.25, 1.5, 2, 4, 6, 8, 10, 12, 24, 36, and 48 h post-dose. Subsequently, the time points were slightly adjusted depending on the pharmacokinetic profile of the 5 mg group of SAD. In other dose groups of SAD, blood samples were collected at the following time points: 0 (pre-dose), 0.33, 0.66, 1, 1.33, 1.66, 2, 3, 4, 6, 8, 10, 12, 24, 36, and 48 h post-dose. Urine samples were collected within 2 h before dosing and during the intervals 0-4 h, 4-8 h, 8-12 h, 12-24 h and 24-48 h after dosing.

In MAD, blood samples were collected at the following time points: 0 (pre-dose), 0.33, 0.66, 1, 1.33, 1.66, 2, 3, 4, 6, 8, 10, 12 h post-dose at Day 1 and 0 (pre-dose), 0.33, 0.66, 1, 1.33, 1.66, 2, 3, 4, 6, 8, 10, 12, 24, 36, 48 h post-dose at Day 8, in addition, 0 h (pre-dose) at Day 5–7 for determining the steady-state. In the 90 mg QD group, 24 h post-dose at Day 1 should be collected.

The blood samples were separated by centrifugation at 4°C, 1,500 g for 10 min. The separated plasma was divided into two centrifuge tubes (each with at least 0.5 mL of plasma), and stored frozen under −80°C until analysis.

### 2.5 Bioanalytical procedures

The plasma and urine concentrations of HSK16149 were analyzed by validated liquid chromatography-tandem mass spectrometry (LC-MS/MS) method in Shanghai Xuhui Central Hospital. The LC-MS/MS system includes a Triple Quad 6500+ mass spectrometer (AB SCIEX, USA) and a liquid chromatograph waters I class system (Waters, USA). HSK16149-D_4_ was chosen as the internal standard. For plasma concentration detection, the lower limit of quantification (LLOQ) was 3 ng/mL, the linear detection range was 3 ng/mL to 3,000 ng/mL. For urine concentration detection, the LLOQ was 0.2 μg/mL, the linear detection range was 0.2 μg/mL to 200 μg/mL.

### 2.6 Pharmacokinetic assessments

Pharmacokinetic parameters were calculated using PhoenixWinNonlin 8.0 software (Pharsight, Cary, NC, United States). Plasma and urine concentration-time data were analyzed by noncompartmental methods. PK parameters mainly included: maximum observed plasma concentration (C_max_), the maximum plasma concentration at steady-state (C_max,ss_) after multi-dose, the area under the concentration-time curve (AUC), AUC from time zero (pre-dose) to the time of the last measurable concentration (AUC_0-t_), AUC from time zero (pre-dose) to infinity (AUC_0-inf_), the AUC over the dosing interval at steady-state (AUC_ss_), time to maximum plasma concentration (T_max_), time to C_max,ss_ (T_max_), terminal elimination half-life (t_1/2_), apparent distribution volume (Vz/F), clearance rate (CL/F), renal clearance (CL_R_); average retention time from time zero to infinity (MRT_0-inf_), accumulation ratio at steady state (R_ac_), degree of fluctuation (DF) and the cumulative fraction of HSK16149 excreted in urine during each collection interval (Fe). For urine PK parameters, we calculated Ae_0–48h_, which was the cumulative amount of drug excreted into the urine over the 48-h collection interval. CLR(L/h) was the renal clearance index, which was calculated from Ae (mg) ×1,000/AUC_0-t_ (h*ng/mL) and Fe_0–48h_, which was the cumulative fraction of the dose excreted as unchanged parent in urine over the 48-h collection interval.

### 2.7 Statistical analyses

Statistical analysis was performed using SAS Software version 9.4 or above (SAS Institute, Cary, NC, United States). Continuous variables were statistically described using the number of cases, mean, median, standard deviation (SD), minimum values, and maximum values; categorical variables were statistically described using the frequency and percentage of each category. Geometric mean and coefficient of variation (%CV) were also used for applicable plasma concentrations and pharmacokinetic parameters. *p* ≤ 0.05 was considered to be statistically significant. A power model was used to investigate the correlation between PK parameters (AUC, C_max_) and doses. ANOVA model was used to calculate the accumulation index of PK parameters after 8 days of continuous administration.

## 3 Results

### 3.1 Demographic profile

A total of 62 eligible subjects were enrolled in SAD and all of them completed the study per protocol. A total of 60 eligible subjects were enrolled in MAD, 1 subject withdrew from the study after administration due to an AE (Grade 2 of urticaria, this subject was proved to be in placebo group after unblinding), 1 subject withdrew from the study after administration due to protocol deviation (The collected serum and urine samples were included in PK analysis group), and the remaining 58 subjects completed the study. The distribution of subjects is displayed in Figure 1. The demographic profile of all enrolled subjects is demonstrated in Table 1.

**TABLE 1:** Demographic profile of enrolled subjects.

	SAD								MAD						
	5 mg (*n* = 3)	10 mg (*n* = 8)	20 mg (*n* = 8)	40 mg (*n* = 8)	60 mg (*n* = 8)	90 mg (*n* = 8)	120 mg (*n* = 6)	Placebo (*n* = 13)	15 mgBID (*n* = 8)	30 mgBID (*n* = 8)	45 mgBID (*n* = 8)	90 mgQD (*n* = 8)	60 mgBID (*n* = 8)	80 mgBID (*n* = 8)	Placebo (*n* = 12)
Age, years	29.0 (6.0)	27.5 (4.4)	24.9 (5.0)	31.5 (4.5)	28.3 (4.6)	27.9 (3.6)	28.3 (4.8)	28.0 (4.4)	30.0 (3.4)	26.5 (4.0)	32.5 (3.0)	29.6 (1.9)	28 (4.8)	30.0 (6.0)	28.8 (3.7)
Gender Male [n (%)]	3 (100%)	7 (87.5%)	8 (100%)	6 (75.0%)	8 (100%)	8 (100%)	5 (83.3%)	12 (92.3%)	8 (100%)	8 (100%)	8 (100%)	8 (100%)	7 (87.5%)	7 (87.5%)	11 (91.7%)
Female [n (%)]	0	1 (12.5%)	0	2 (25.0%)	0	0	1 (16.7%)	1 (7.7%)	0	0	0	0	1 (12.5%)	1 (12.5%)	1 (8.3%)
Height, cm	169.0 (6.72)	168.9 (5.10)	169.0 (5.86)	166.8 (7.99)	170.4 (5.80)	171.6 (8.75)	166.3 (5.92)	166.3 (6.22)	166.1 (7.65)	166.5 (3.84)	168.0 (3.00)	164.9 (3.93)	170.9 (3.53)	162.5 (6.22)	165.6 (8.47)
Weight, kg	65.8 (12.92)	65.4 (4.74)	66.1 (9.53)	63.5 (7.27)	67.8 (5.69)	66.4 (5.54)	62.9 (7.00)	60.0 (7.05)	61.2 (7.68)	59.1 (5.28)	62.1 (5.01)	62.5 (4.12)	65.9 (8.99)	58.0 (7.47)	64.7 (8.79)
BMI, kg/m^2^	22.87 (2.80)	22.92 (1.35)	23.05 (1.96)	22.84 (1.89)	23.37 (1.99)	22.61 (1.74)	22.7 (1.79)	21.65 (1.44)	22.2 (1.63)	21.3 (1.43)	22.0 (1.69)	23.0 (1.19)	22.6 (2.93)	21.9 (1.84)	23.5 (1.76)

Note: Data are expressed as mean (SD), except for gender, which is shown as n (%).

Abbreviation: BMI, body mass index.

### 3.2 Safety

In SAD, 49 subjects taking HSK16149, of which 28 subjects (57.1%) had TEAEs (Table 2) and 23 subjects (46.9%) (Table 2) had TEAEs judged to be related to the study drug. 13 subjects taking placebo, of which 5 subjects (38.5%) (Table 2) had TEAEs. Among the reported TEAEs after HSK16149 administration, 2 cases with CTCAE Grade 3 in the 120 mg group were considered to be related to the study drug, 1 case in the 60 mg group, and 1 case in the 90 mg group with CTCAE Grade 2 were considered to be related to the study drug. The rest are grade 1 AEs. The most common drug-related AEs were somnolence and dizziness. No SAEs occurred throughout the study, and no subject withdrew from the study due to AE.

**TABLE 2:** Treatment-emergent adverse events (TEAEs) in SAD.

	5 mg	10 mg	20 mg	40 mg	60 mg	90 mg	120 mg	Placebo	Overall
	(*n* = 3)	(*n* = 8)	(*n* = 8)	(*n* = 8)	(*n* = 8)	(*n* = 8)	(*n* = 6)	(*n* = 13)	(*n* = 62)
	n (%)	n (%)	n (%)	n (%)	n (%)	n (%)	n (%)	n (%)	n (%)
TEAE	1 (33.3%)	2 (25.0%)	4 (50.0%)	1 (12.5%)	8 (100%)	7 (87.5%)	5 (83.3%)	5 (38.5%)	33 (53.2%)
Drug related AE	0	1 (12.5%)	3 (37.5%)	1 (12.5%)	7 (87.5%)	7 (87.5%)	4 (66.7%)	4 (30.8%)	27 (43.5%)
CNS related AE	0	0.00%	0.00%	0.00%	7 (87.5%)	6 (75.0%)	4 (66.7%)	3 (23.1%)	20 (32.3%)
Dizziness	0	0	0	0	7 (87.5%)	4 (50.0%)	4 (66.7%)	3 (23.1%)	18 (29.0%)
Somnolence	0	0	0	0	2 (25.0%)	6 (75.0%)	2 (33.3%)	0	10 (16.1%)
Ataxia	0	0	0	0	0	0	2 (33.3%)	0	2 (3.2%)
Headache	0	0	0	0	0	0	2 (33.3%)	0	2 (3.2%)
Hyporeflexia	0	0	0	0	0	0	1 (16.7%)	0	1 (1.6%)
Hypesthesia	0	0	0	0	0	0	1 (16.7%)	0	1 (1.6%)
Microscopic Hematuria	0	0	3 (37.5%)	0	0	0	0	0	3 (4.8%)
Uric acid Elevated	1 (33.3%)	0	1 (12.5%)	0	1 (12.5%)	0	0	0	3 (4.8%)
Direct bilirubin elevation	0	0	0	0	0	0	1 (16.7%)	0	1 (1.6%)
Urine glucose	0	1 (12.5%)	0	0	0	0	0	0	1 (1.6%)
Blood glucose elevation	0	1 (12.5%)	0	0	0	0	0	0	1 (1.6%)
High blood pressure	0	0	0	0	1 (12.5%)	0	0	0	1 (1.6%)
Urine white blood cell positive	0	0	0	0	0	0	0	1 (7.7%)	1 (1.6%)
Nausea	0	0	0	0	0	1 (12.5%)	2 (33.3%)	0	3 (4.8%)
Diarrhea	0	1 (12.5%)	0	1 (12.5%)	0	0	1 (16.7%)	0	3 (4.8%)
Dry mouth	0	0	0	0	0	1 (12.5%)	2 (33.3%)	0	3 (4.8%)
Bloating	0	0	0	0	0	0	1 (16.7%)	0	1 (1.6%)
Vomiting	0	0	0	0	0	0	1 (16.7%)	0	1 (1.6%)
Swallow hard	0	0	0	0	0	0	1 (16.7%)	0	1 (1.6%)
Fever	0	1 (12.5%)	0	0	0	0	0	0	1 (1.6%)
Fatigue	0	0	0	0	1 (12.5%)	0	0	0	1 (1.6%)
Foreign body sensation	0	0	0	0	0	0	1 (16.7%)	0	1 (1.6%)
Diplopia	0	0	0	0	0	0	2 (33.3%)	0	2 (3.2%)
Blurred vision	0	0	0	0	0	1 (12.5%)	1 (16.7%)	0	2 (3.2%)
Dysuresia	0	0	0	0	0	0	2 (33.3%)	0	2 (3.2%)
Pharyngitis	0	0	0	0	0	0	1 (16.7%)	0	1 (1.6%)
Dorsalgia	0	0	1 (12.5%)	0	0	0	0	0	1 (1.6%)
Oropharyngeal pain	0	0	0	0	0	1 (12.5%)	0	0	1 (1.6%)
Intraventricular conduction defect	0	0	0	0	0	1 (12.5%)	0	0	1 (1.6%)
Peeling skin	0	0	0	0	0	0	0	1 (7.7%)	1 (1.6%)

Abbreviations: TEAE, treatment-emergent adverse event.

In MAD, 48 subjects taking HSK16149, of which 44 subjects (91.7%) had TEAEs (Table 3), and 43 subjects (89.6%) (Table 3) had TEAEs judged to be related to the study drug; 12 subjects taking placebo, of which 11 subjects (91.7%) had TEAEs (Table 3). Among the reported TEAEs after HSK16149 administration, 1 case in the 30 mg BID group and 1 case in the 45 mg BID group with CTCAE Grade 2 were considered to be related to the study drug, 1 case in the 90 mg QD group with CTCAE Grade 2 was not considered to be related to the study drug, 1 subject in the placebo group withdrew from the study due to urticaria, the rest are grade 1 AEs. The most common drug-related AEs were dizziness, elevated blood triglycerides, and diarrhea. No AEs of grade 3 or higher occurred throughout the study.

**TABLE 3:** Treatment-emergent adverse events (TEAEs) in MAD.

	15 mg BID	30 mg BID	45 mg BID	90 mg QD	60 mg BID	80 mg BID	Placebo	Overall
	(*n* = 8)	(*n* = 8)	(*n* = 8)	(*n* = 8)	(*n* = 8)	(*n* = 8)	(*n* = 12)	(*n* = 60)
	n (%)	n (%)	n (%)	n (%)	n (%)	n (%)	n (%)	n (%)
TEAE	7 (87.5%)	7 (87.5%)	7 (87.5%)	8 (100%)	7 (87.5%)	8 (100%)	11 (91.7%)	55 (91.7%)
Drug related AE	7 (87.5%)	6 (75.0%)	7 (87.5%)	8 (100%)	7 (87.5%)	8 (100%)	8 (66.7%)	51 (85.0%)
CNS related AE	1 (12.5%)	3 (37.5%)	6 (75.0%)	6 (75.0%)	7 (87.5%)	8 (100%)	3 (25.0%)	34 (56.7%)
Dizziness	1 (12.5%)	3 (37.5%)	6 (75.0%)	6 (75.0%)	7 (87.5%)	8 (100%)	3 (25.0%)	34 (56.7%)
Somnolence	0	0	0	0	0	2 (25.0%)	0	2 (3.3%)
Headache	1 (12.5%)	0	0	0	0	0	0	1 (1.7%)
Nausea	1 (12.5%)	0	0	1 (12.5%)	1 (12.5%)	3 (37.5%)	0	6 (10.0%)
Diarrhea	4 (50.0%)	0	1 (12.5%)	0	0	1 (12.5%)	1 (8.3%)	7 (11.7%)
Dry mouth	0	0	1 (12.5%)	1 (12.5%)	1 (12.5%)	1 (12.5%)	1 (8.3%)	5 (8.3%)
Canker sore	3 (37.5%)	0	1 (12.5%)	0	0	0	0	4 (6.7%)
Constipation	1 (12.5%)	2 (25.0%)	0	0	0	0	0	3 (5.0%)
Stomachache	2 (25.0%)	0	0	1 (12.5%)	0	0	1 (8.3%)	4 (6.7%)
Abdominal distension	2 (25.0%)	0	0	0	0	0	0	2 (3.3%)
Cheilitis	0	1 (12.5%)	0	0	0	0	0	1 (1.7%)
Oral mucositis	0	0	0	1 (12.5%)	0	0	0	1 (1.7%)
Vomiting	0	0	0	1 (12.5%)	0	0	0	1 (1.7%)
Toothache	0	0	0	0	0	0	2 (16.7%)	2 (3.3%)
Hypertriglyceridemia	2 (25.0%)	1 (12.5%)	2 (25.0%)	1 (12.5%)	1 (12.5%)	2 (25.0%)	0	9 (15.0%)
Hematuric acid elevated	1 (12.5%)	0	3 (37.5%)	1 (12.5%)	0	0	2 (16.7%)	7 (11.7%)
Alanine aminotransferase elevated	0	0	1 (12.5%)	0	1 (12.5%)	1 (12.5%)	1 (8.3%)	4 (6.7%)
γ-glutamyltransferase elevated	0	0	1 (12.5%)	0	0	0	1 (8.3%)	2 (3.3%)
Aspartate aminotransferase elevated	0	0	0	0	0	1 (12.5%)	0	1 (1.7%)
Creatine Phosphokinase elevated	0	0	0	1 (12.5%)	0	0	0	1 (1.7%)
Urine white blood cell positive	0	0	0	0	0	0	2 (16.7%)	2 (3.3%)
Creatine phosphokinase MB increased	0	0	0	0	0	0	1 (8.3%)	1 (1.7%)
Fatigue	0	0	0	1 (12.5%)	0	1 (12.5%)	0	2 (3.3%)
Chest pain	0	1 (12.5%)	0	0	0	1 (12.5%)	0	2 (3.3%)
Dysuresia	0	1 (12.5%)	0	0	1 (12.5%)	0	0	2 (3.3%)
Hematuria	1 (12.5%)	0	1 (12.5%)	0	0	0	1 (8.3%)	3 (5.0%)
Skin erosion	0	1 (12.5%)	0	0	0	0	0	1 (1.7%)
Papule	0	0	1 (12.5%)	0	0	0	0	1 (1.7%)
Eczema	1 (12.5%)	0	0	0	0	0	0	1 (1.7%)
Pruritus	0	1 (12.5%)	0	0	0	0	0	1 (1.7%)
Urticaria	0	0	0	0	0	0	1 (8.3%)	1 (1.7%)
Lung infection	0	1 (12.5%)	0	0	0	0	0	1 (1.7%)
Upper respiratory tract infection	0	0	1 (12.5%)	0	0	0	0	1 (1.7%)
Nasal obstruction	0	0	1 (12.5%)	0	0	0	1 (8.3%)	2 (3.3%)
Run at the nose	0	0	0	0	0	0	1 (8.3%)	1 (1.7%)
Papular urticaria	0	0	0	1 (12.5%)	0	0	0	1 (1.7%)
Arthralgia	0	0	0	0	0	0	1 (8.3%)	1 (1.7%)
Limb pain	0	0	0	0	0	0	1 (8.3%)	1 (1.7%)

Abbreviations: TEAE, treatment-emergent adverse event; BID, twice daily.

### 3.3 Pharmacokinetic properties

**FIGURE 2:**
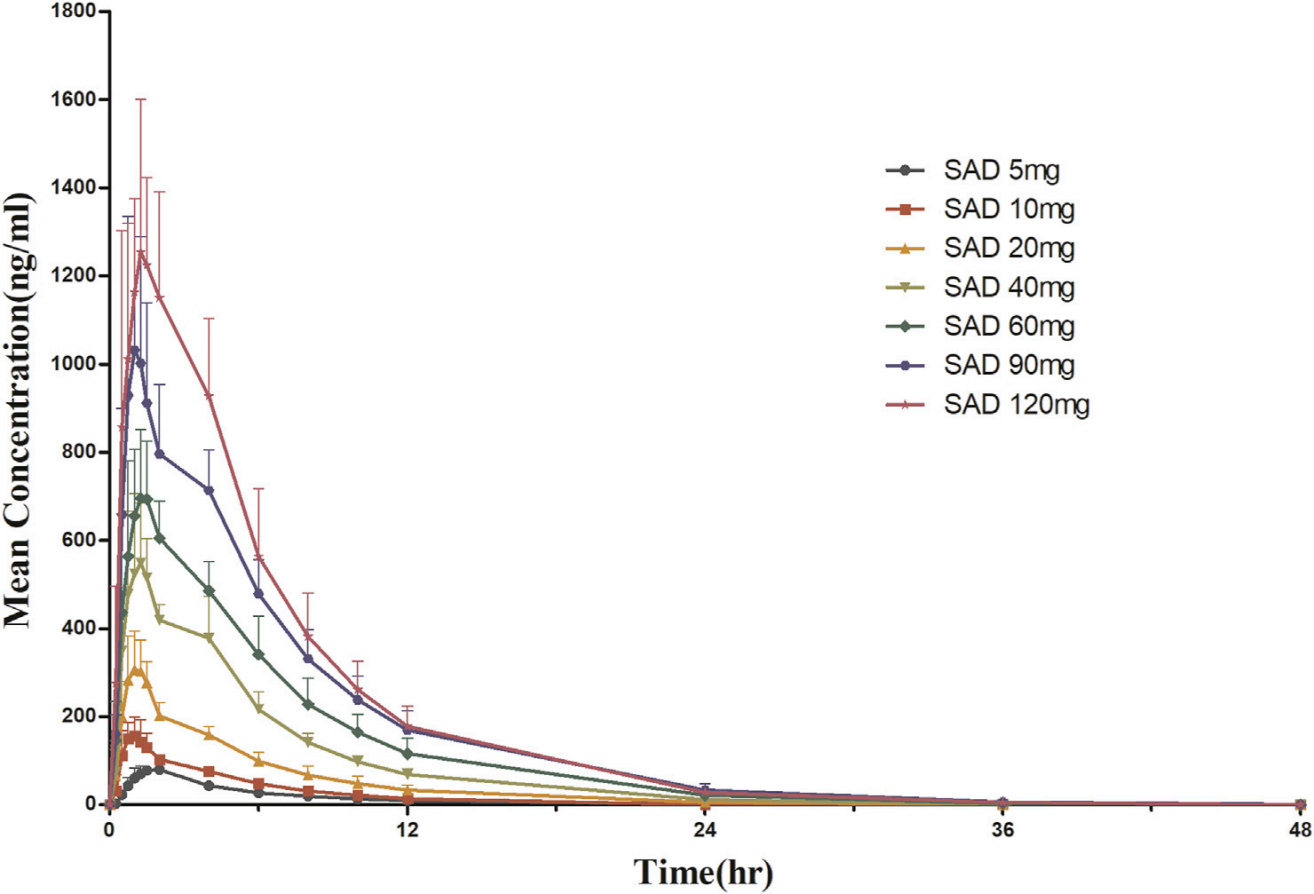
Mean plasma concentration-time curves after a single dose of HSK16149.

For SAD: The main plasma PK parameters in each dose group after a single dose of HSK16149 are summarized in Table 4, and the mean plasma drug concentration-time curves are shown in Figure 2.

**TABLE 4:** Main plasma and urinary PK Parameters in SAD.

Pharmacokinetic paramaeters	5 mg (*n* = 3)	10 mg (*n* = 8)	20 mg (*n* = 8)	40 mg (*n* = 8)	60 mg (*n* = 8)	90 mg (*n* = 8)	120 mg (*n* = 6)
Plasma parameters							
C_max_ (ng/mL)	81.84 (4.05)	183.26 (42.42)	330.89 (75.03)	598.10 (145.38)	762.45 (128.09)	1172.19 (344.44)	1426.95 (270.70)
T_max_ (h)	1.6 (0.4)	1.5 (0.7)	1.5 (0.4)	1.9 (0.9)	1.7 (0.6)	1.7 (0.6)	1.7 (0.8)
t_1/2_ (h)	4.4 (0.5)	3.7 (0.4)	4.3 (0.7)	5.3 (1.8)	5.6 (3.0)	5.3 (1.7)	6.4 (4.1)
AUC_0-inf_ (h*ng/mL)	471.0 (24.6)	797.1 (102.7)	1734.8 (270.0)	3547.3 (409.7)	5217.5 (788.2)	7503.8 (674.2)	8952.0 (1700.5)
AUC_0-t_ (h*ng/mL)	461.7 (25.7)	718.3 (87.5)	1690.3 (258.2)	3479.6 (421.3)	5159.3 (789.2)	7449.8 (677.5)	8909.3 (1712.8)
V_z_/F (mL)	67194.0 (9779.3)	67352.8 (8754.8)	71949.0 (12833.3)	88465.0 (39157.4)	100483.3 (76136.4)	91850.3 (27960.0)	132939.0 (99813.7)
Cl/F (mL/h)	10639.3 (566.6)	12728.3 (1621.4)	11758.5 (1695.7)	11415.0 (1375.7)	11755.8 (1956.0)	12077.4 (1061.6)	13836.7 (2778.8)
MRT_0-inf_ (h)	6.0 (0.5)	5.2 (0.6)	5.8 (1.1)	6.2 (0.7)	6.8 (1.0)	7.0 (1.3)	6.4 (0.3)
Urinary parameters							
Ae_0–48h_ (mg)	3.96 (0.1572)	7.833 (0.5926)	16.708 (1.6223)	31.686 (4.1301)	48.135 (5.7587)	63.765 (5.8457)	88.457 (18.0779)
CL_R_ (L/h)	8.603 (0.7015)	10.998 (1.1341)	10.063 (1.6328)	9.175 (1.3491)	9.439 (1.22)	8.645 (1.2836)	9.957 (1.4256)
Fe_0–48h_ (%)	79.23 (3.086)	78.33 (5.926)	83.53 (8.106)	79.21 (10.319)	80.23 (9.596)	70.84 (6.49)	73.73 (15.059)

Notes: Data are expressed as mean (SD), except for T_max_, which is shown as median (min, max). **p < 0.01: The difference of the PK parameter among different dose groups is considered to be statistically significant.

Abbreviations: C_max_, maximum observed plasma concentration; T_max_, time to maximum plasma concentration; t_1/2_, terminal elimination half-life; AUC_0-inf_, the area under the concentration-time curve from time zero to infinity; AUC_0-t_, the area under the concentration-time curve from time zero to the time of the last measurable concentration; V_z_/F, apparent distribution volume; Cl/F, clearance rate; CL_R(L/h)_, the renal clearance index; MRT_0-inf_, average retention time from zero to infinity; Ae_0–48_ _h_, cumulative amount of drug excreted into the urine over the 48-h collection interval; Fe_0–48_ _h_, cumulative fraction of the dose excreted as unchanged parent in urine over the 48-h collection interval.

The Vz/F, Cl/F, and MRT_0-inf_ of the 7 dose groups were similar (*p* > 0.05), while the parameters AUC and C_max_ were dose proportional. HSK16149 was rapidly absorbed, T_max_ occurred at 1.5-1.7 h and t_1/2_ ranged from 3.7 to 6.4 h. The PK parameters of HSK16149 in urine are shown in Table 4. The cumulative amount of HSK16149 Ae_0–48 h_ increased with the dosage increment, the average renal clearance was consistent in different dose groups. The range of Fe_0–48 h_ in different dose groups was 70.84%-83.53% which means HSK16149 was mainly eliminated via urinary excretion.

**FIGURE 3:**
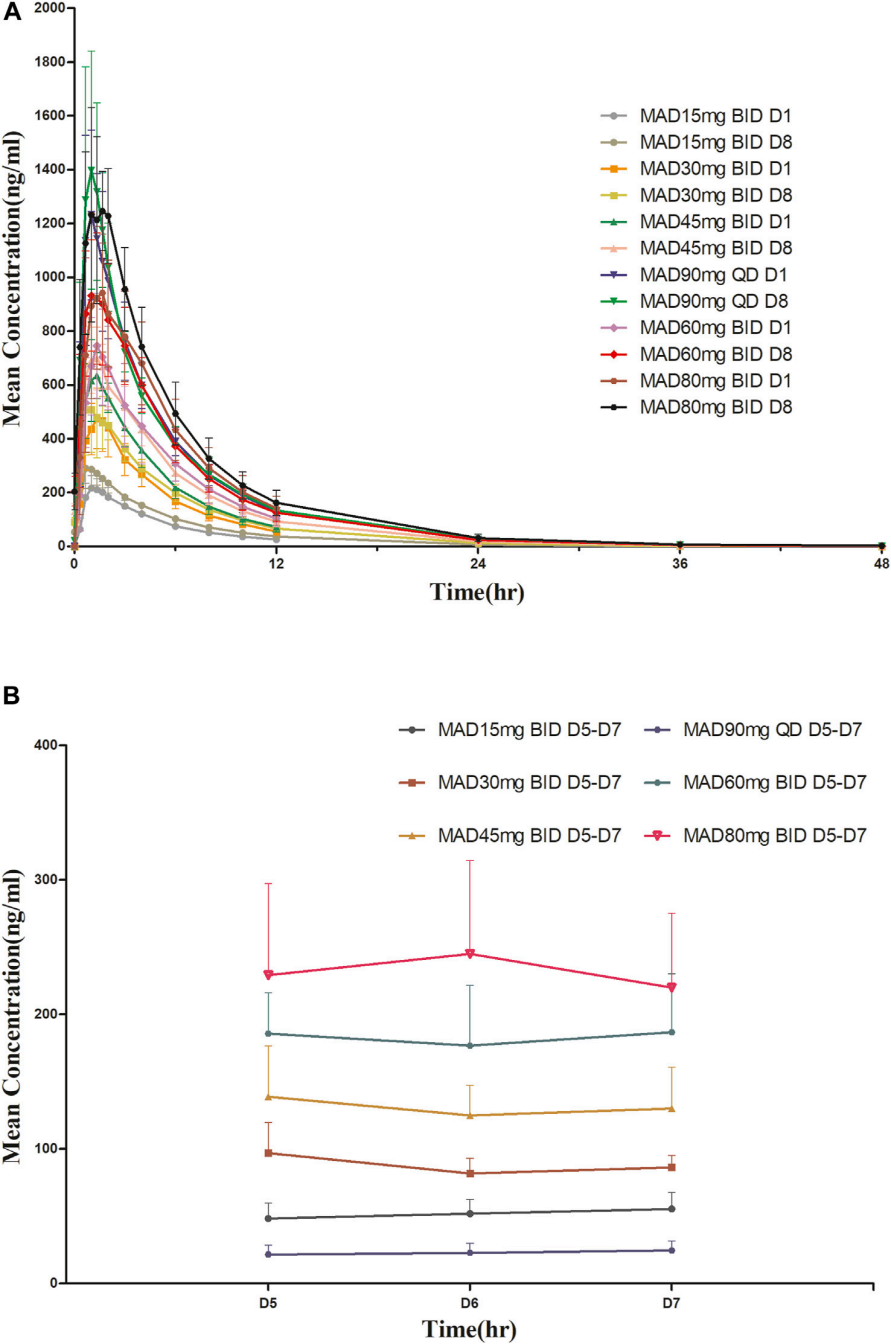
Mean plasma concentration-time profiles after multiple doses of HSK16149. **(A)** HSK16149 15-80 mg BID and 90 mg QD at D1, D8. **(B)** HSK16149 15-80 mg BID and 90 mg QD at D5, D6, D7.

For MAD: The main PK parameters of HSK16149 for MAD are presented in Table 5. The mean plasma drug concentration-time curves at D1 and D8 are demonstrated in Figure 3.

**TABLE 5:** Main plasma PK parameters in MAD.

Time	Pharmacokinetic paramaeters	15 mg BID (*n* = 8)	30 mg BID (*n* = 8)	45 mg BID (*n* = 8)	90 mg QD (*n* = 8)	60 mg BID (*n* = 8)	80 mgBID (*n* = 8)
D1	T_max_ (h)	1.1 (0.4)	1.4 (0.5)	1.1 (0.4)	1.0 (0.4)	1.4 (0.3)	1.7 (1.0)
	t_1/2_ (h)	4.0 (0.4)	3.8 (0.5)	3.7 (0.2)	4.8 (0.6)	3.9 (0.5)	3.8 (0.4)
	MRT_0-inf_ (h)	5.8 (0.4)	5.6 (0.7)	5.4 (0.6)	6.0 (0.7)	6.1 (0.5)	5.9 (0.7)
	C_max_ (ng/mL)	230.15 (36.73)	508.08 (117.16)	678.70 (121.25)	1315.7 (312.3)	788.7 (172.7)	1047.6 (274.9)
	AUC_0-inf_ (h*ng/mL)	1249.1 (174.1)	2748.8 (304.8)	3621.3 (448.8)	6892.5 (768.0)	4622.5 (1012.8)	6413.8 (1352.8)
	AUC_0-t_ (h*ng/mL)	1094.9 (135.2)	2440.0 (309.6)	3230.0 (339.9)	6727.5 (792.6)	4020.0 (837.8)	5632.5 (1109.8)
	V_z_/F (mL)	70200.0 (6656.9)	60912.5 (13367.8)	66837.5 (6030.6)	91125.0 (18441.8)	75912.5 (14640.1)	69775.0 (15959.7)
	Cl/F (mL/h)	12220.0 (1695.1)	11037.5 (1301.6)	12600.0 (1657.9)	13212.5 (1565.2)	13533.8 (2853.4)	13013.8 (2925.3)
D8	T_max_ (h)	0.8 (0.3)	1.3 (0.6)	1.3 (0.4)	1.0 (0.4)	1.3 (0.7)	1.7 (1.0)
	t_1/2_ (h)	4.8 (0.5)	5.1 (0.5)	7.0 (3.7)	7.0 (3.1)	5.9 (1.8)	3. (0.4)
	MRT_0-inf_ (h)	6.6 (0.9)	6.6 (0.7)	6.8 (0.8)	6.1 (0.8)	6.6 (0.5)	5.9 (0.7)
	C_ss_min_ (ng/mL)	37.09 (10.86)	64.80 (6.67)	93.32 (19.856)	20.88 (7.06)	125.88 (33.81)	159.59 (48.60)
	C_ss_max_ (ng/mL)	311.71 (48.16)	575.75 (117.8)	724.68 (107.45)	1504.88 (357.18)	983.71 (224.23)	1396.88 (276.41)
	AUC_0-inf,ss_ (h*ng/mL)	1806.3 (340.5)	3367.5 (338.43)	4735.0 (666.48)	7228.8 (885.9)	6466.3 (1376.3)	8502.5 (1264.0)
	AUC_0-t,ss_ (h*ng/mL)	1765.0 (336.4)	3296.3 (353.23)	4668.8 (666.4)	7155.0 (896.6)	6413.8 (1379.4)	8435.0 (1279.9)
	Vz/F_ss_(mL)	71287.5 (8243.9)	80187.5 (11908.9)	117112.5 (64277.8)	130525.0 (56342.8)	101000.0 (47533.0)	123262.5 (79993.6)
	CL/F_ss_ (mL/h)	10295.0 (1619.6)	10855 (1320.03)	11762.5 (1313.6)	13050.0 (1434.3)	11686.3 (2551.3)	11581.3 (1580.7)
	Rac (AUC_0-inf_)	1.44 (0.15)	1.23 (0.08)	1.31 (0.13)	1.05 (0.12)	1.4 (0.08)	1.35 (0.16)
	Rac (C_max_)	1.37 (0.22)	1.15 (0.16)	1.09 (0.21)	1.17 (0.24)	1.26 (0.19)	1.39 (0.35)

Notes: Data are expressed as mean (SD), except for T_max_, which is shown as median (min, max). **p < 0.01: The difference of the PK parameter among different dose groups is considered to be statistically significant.

Abbreviations: T_max_, time to maximum plasma concentration; t_1/2,_ terminal elimination half-life; MRT_0-inf,_ average retention time from zero to infinity; C_max_, maximum observed plasma concentration; AUC_0-inf,_ area under the concentration-time curve from time zero to infinity; AUC_0-t,_ area under the concentration-time curve from time zero to the time of the last measurable concentration; Vz/F, apparent distribution volume; Cl/F, apparent total body clearance after oral administration; C_ss_min,_ minimum observed plasma concentration (at steady state); C_ss_max,_ maximum observed plasma concentration (at steady state); AUC_0-inf_,_ss,_ area under the concentration-time curve from time zero to infinity (at steady state); AUC_0-t,ss,_ area under the concentration-time curve from time zero to the time of the last measurable concentration (at steady state); Vz/F_ss,_ apparent distribution volume (at steady state); CL/F_ss,_ apparent total body clearance after oral administration (at steady state); Rac, accumulation ratio at steady state.

The AUC and C_max_ were almost dose-proportional. The average clearance rate and distribution volume of HSK16149 at all dose levels were comparable. The mean t_1/2_ of HSK16149 ranged from 3.7 to 7.1 h, and R_ac_ ranged from 1.04 to 1.35, indicating a slight accumulation on day 8.

## 4 Discussion

This article describes the first-in-human study of HSK16149, a novel, potent GABA analog for the treatment of neuropathic pain. The study shows the pharmacokinetics of HSK16149 in healthy Chinese subjects, and the safety results indicate that HSK16149 was well tolerated.

The dose selection rationale was based on a comprehensive evaluation of the NOAEL/MRSD approach, the drug formulation specifications of HSK16149, and the approved dosage of Pregabalin. In the preclinical repeated dose toxicity study in rats, the no-observed adverse effect level (NOAEL) dose was 120 mg/kg, the corresponding human equivalent dose as 1,200 mg, therefore, the maximum recommended starting dose (MRSD) was 120 mg (with the safety factor of 10). In the preclinical repeated dose toxicity study in cynomolgus monkeys, the maximum recommended starting dose (MRSD) was 30 mg (with the safety factor of 10), and according to the phase II reproductive toxicity tests, the MRSD was 19.4 mg. At the same time, considering the safety of subjects and the drug formulation specifications of HSK16149 (5 mg and 20 mg), the initial dose of HSK16149 in this first-in-human phase I study was finally set at 5 mg. In a preclinical single dose toxicity study in rats, the maximum toxicity dose (MTD) in human was calculated to be 644 mg (with the safety factor of 10). In preclinical pharmacology studies, the efficacy of HSK16149 was approximately 2–3 times better than that of pregabalin, with an approved dosage of 75 or 150 mg twice a day for pregabalin. Therefore, it was estimated that the clinically proposed dosage of HSK16149 may be 75 mg. Taking the above situations into account and the drug formulation specifications, the maximum dose set for this study was 120 mg.

CNS-related TEAEs were the most common TEAEs in both SAD and MAD. Based on the mechanism of action of HSK16149, it was not unexpected that CNS-related TEAEs were the most commonly reported TEAEs. The CNS-related TEAEs are consistent with pregabalin and mirogabalin, subjects developed tolerance to these TEAEs (Bhusal et al., 2016; Brown et al., 2018; Li et al., 2023). During the study, all observed CNS-related TEAEs were tolerated. In SAD, the duration of CNS-related TEAEs was 4 h to 3 days; In MAD, except for a CNS-related TEAE with the longest duration of 7 days in one subject and a CNS-related TEAE with a duration of 6 days in one subject, all other subjects experienced CNS-related TEAEs with a duration of 10 min to 1 day. The duration of HSK16149 CNS-related TEAE was similar to mirogabalin, which required 4-5 days to get resolved or improved (Brown et al., 2018; Li et al., 2023). Except for one subject with grade 3 TEAE in the 120 mg group who was treated with an intravenous drip of 10% potassium chloride + 0.9% saline during the study, most CNS-related TEAEs can recovered spontanously without treatment. Majority subjects only experienced 1–2 times of CNS-related TEAEs during the MAD study. Gastrointestinal-related TEAEs were also found in both SAD and MAD, which were also reported as side effects of pregabalin (Bhusal et al., 2016). Almost all TEAEs were mild to moderate, except 1 subject taking placebo discontinued from the study due to urticarial.

Pharmacokinetic results showed that the AUC of SAD (5-120 mg) and MAD (15-90 mg) was proportional to the dose. If the criterion is relaxed to (0.5, 2.0), the proportional relationship between C_max_ and dose is also established. The mean value of Fe in the urine of each dose group within 48 h after HSK16149 administration was between 70.84% and 83.53%, indicating that HSK16149 was mainly excreted through urine. As HSK16149 can be taken with or without food, which was supported by the food effect study conducted by the authors, HSK16149 capsules were still administered under fasting conditions in the MAD study to maintain consistency with the previous SAD study. The exposure of the first dose of the 45 mg BID group (*n* = 8, C_max_ = 678.70 ± 121.25 ng/mL, AUC_0-inf_ = 3621.3 ± 448.8 h*ng/mL) in the MAD study was consistent with that of the food impact study (Fasted group: dose = 45 mg, *n* = 25, C_max_ = 676.29 ± 193.086 ng/mL, AUC_0-inf_ = 4074.4 ± 705.42 h*ng/mL) (Wu et al., 2023). Similarly, the C_max_ and AUC of the 40 mg group in the SAD study were slightly lower than those observed in the Food Effect Study (Wu et al., 2023).

We evaluated different dosing frequencies in MAD, and the results showed that the AUC_ss_ of the 45 mg BID group and the 90 mg QD group were similar in drug exposure. The T_max_ of the 45 mg BID group and the 90 mg QD group were 1.25 h vs. 1.00 h, respectively; the t_1/2_ of the two dose groups were close, 6.95 h vs. 7.00 h, respectively; the AUC accumulation index in the 45 mg BID group was slightly greater than that in the 90 mg QD group, the AUC_0-inf_ accumulation index and the C_max_ accumulation index were 1.31 vs. 1.05, 1.09 vs. 1.17, respectively. Preclinical *in vitro* pharmacological studies showed that the IC_90_ of HSK16149 for the inhibition of calcium channel α2δ subunit was about 100 nM, with an estimated human exposure level of 36.746 ng/mL (Gou et al., 2021). In the 45 mg BID group, the mean C_ss_min_ was 93.32 ng/mL, higher than the human exposure level of 36.746 ng/mL estimated by IC_90_, while the mean C_ss_min_ of the 90 mg QD group was 20.88 ng/mL (<36.746 ng/mL), indicating that the concentration of 45 mg BID group higher than the effective concentrations simulated by preclinical data. The 45 mg BID group and the 90 mg QD group had good safety, and the incidence of TEAE in the 45 mg BID group was lower than that in the 90 mg QD group. According to the PK and safety data, BID administration has more advantages in subsequent clinical studies.

Based on these results, HSK16149 was safe and generally well tolerated. The trial not only assessed the tolerability of the drug in humans after single and multiple administrations, but also assessed the pharmacokinetics and safety of different administration frequencies. We recommend that the maximum proposed dose for further clinical studies should not exceed 80 mg BID. Phase III trial is underway to evaluate the efficacy and safety of HSK16149 in patients with postherpetic neuralgia (CTR20213431).

## 5 Limitations

One limitation of our study is that, as a phase I study, the number of subjects in each group was relatively small and the maximum age was 45 years old, further research is needed to fully evaluate the safety and tolerability of this drug. In addition, only 8 female subjects (7 in HSK16149 and 1 in placebo) enrolled in the study, and more female subjects need to be recruited to further investigate gender differences in pharmacokinetics.

## 6 Conclusions

HSK16149 was well tolerated in healthy Chinese subjects with a single dose range of 5–120 mg and multiple doses range of 15–80 mg twice daily. These results support the dose selection and further clinical evaluation of HSK16149 for further clinical studies. Based on the safety and pharmacokinetic data, 80 mg twice daily (BID) was suggested as the highest target dose for further clinical development.

## Data Availability

The original contributions presented in the study are included in the article/supplementary material, further inquiries can be directed to the corresponding author.
